# Geographic variation in caesarean delivery in India

**DOI:** 10.1111/ppe.12807

**Published:** 2021-08-31

**Authors:** Justin Rodgers, Hwa‐Young Lee, Rockli Kim, Nachiket Mor, S. V. Subramanian

**Affiliations:** ^1^ Center for Population and Development Studies Harvard University Cambridge MA USA; ^2^ Department of Global Health and Population Harvard T.H. Chan School of Public Health Boston MA USA; ^3^ Institute of Convergence Science (ICONS), Convergence Science Academy Yonsei University Seoul Korea; ^4^ Division of Health Policy and Management, College of Health Sciences Korea University Seoul Korea; ^5^ Interdisciplinary Program in Precision Public Health Department of Public Health Sciences Graduate School of Korea University Seoul Korea; ^6^ The Banyan Academy of Leadership in Mental Health Thiruvidandai India; ^7^ Department of Social and Behavioral Sciences Harvard T.H. Chan School of Public Health Boston MA USA

**Keywords:** caesarean, geography, global health, India, multilevel modelling, variation

## Abstract

**Background:**

The rate of caesarean delivery has increased markedly both globally and within India. However, there is considerable variation within countries. No previous studies have examined the relative importance of multiple geographic levels in shaping the distribution of caesarean delivery and to what extent they can be explained by individual‐level risk factors.

**Objectives:**

To describe geographic variation in caesarean delivery and quantify the contribution of individual‐level risk factors to the variation in India.

**Methods:**

We conducted four‐level logistic regression analysis to partition total variation in caesarean delivery to three geographic levels (states, districts and communities) and quantify the extent to which variance at each level was explained by a set of 20 sociodemographic, medical and institutional risk factors. Stratified analyses were conducted by the type of delivery facility (public/private).

**Results:**

Overall prevalence of caesarean delivery was 19.3% in India in 2016. Most geographic variation was attributable to states (44%), followed by communities (32%), and lastly districts (24%). Adjustment for all risk factors explained 44%, 52% and 46% of variance for states, districts and communities, respectively. The proportion explained by individual risk factors was larger in public facilities than in private facilities at all three levels. A substantial proportion of between‐population variation still existed even after clustering of individual risk factors was comprehensively adjusted for.

**Conclusions:**

Diverse contextual factors driving high or low rate of caesarean delivery at each geographic level should be explored in future studies so that tailored intervention can be implemented to reduce the overall variation in caesarean delivery.

**Synopsis**

**Study question**

What is the extent of geographic variation in caesarean delivery in India? Does this differ by geographic level and by type of delivery facility? How much are these trends in geographic variation driven by clustering of individual risk factors?

**What's already known**

There is preliminary evidence suggesting a meaningful degree of variation in caesarean delivery in India, but details are unclear.

**What this study adds**

This study provides further evidence on the considerable between‐population disparities in caesarean delivery in India. Specifically, that variation in caesarean delivery is especially prominent across states and within public facilities. Further, variation across all three geographic levels was partially explained by clustering of sociodemographic, medical and institutional risk factors, albeit to varying degrees.

## 1 INTRODUCTION

Caesarean delivery is an essential component of obstetric care that can be a life‐saving intervention for mothers and infants when its use is medically justified.^1^ On the other hand, medically unnecessary caesarean surgery may produce additional short‐term and long‐term health risks for women and newborns.^2^ While the World Health Organization (WHO) has recommended an optimal country‐level caesarean delivery rate of approximately 10%–15% at population level,^3^ caesarean delivery rates have continued to rise throughout the twenty‐first century, expanding from a global average of 12.1% in 2000 to 21.1% in 2015.^4^ India has also seen a steady rise in the use of caesarean deliveries as in the global trend, with the national rate more than doubling from 7.1% in 2006 to 17.2% in 2016.^5^, ^6^ However, an exclusive focus on country‐level averages often masks large within‐country variations. For example, the India National Family Health Survey (NFHS) 2016 recorded a caesarean delivery rate of 57.7% in the state of Telangana, but only 5.8% in the state of Nagaland.^5^

Planning and management of the healthcare system in India revolves around several important geographic levels. Firstly, India is composed of 36 states and union territories, each of which has its own publicly funded healthcare infrastructure.^7^ Basically, state governments support payments only for services provided by public providers. Services in private facilities are paid by patients' out‐of‐pocket expenditures.^7^ The next administrative unit is the district level, which takes charge of distributing healthcare and public health services to each of the 640 districts in India.^8^, ^9^ Finally, at the community level, there are community health workers who engage in activities to link the multitude of communities across India to the formal healthcare system, often addressing diverse social and cultural barriers.^10^

Although a number of factors ‐ both internal and external to the healthcare system ‐ operate across multiple geographic levels to shape variation in caesarean delivery rates across India, prior studies are mostly based on a single‐level or, at most, a two‐level analysis of population means, and therefore missed important aspects of this context.^11^, ^12^, ^13^, ^14^, ^15^, ^16^, ^17^, ^18^ Relying only on a single geographic level at a time often leads to a ‘missing unit problem’—that is, the relative importance of any given unit can only be truly estimated when all levels that are thought to influence the outcome are simultaneously considered, and therefore, incomplete consideration of all geographic scales may lead to over‐ or underestimation of their importance.^19^ Additionally, few studies have examined the extent to which clustering of correlated risk factors may explain variations in caesarean delivery. As described in previous studies, a number of individual determinants may affect the prevalence of caesarean delivery including sociodemographic, health, medical and institutional factors, which may be differentially dispersed across the multiple geographic levels of India.^11^, ^12^, ^13^, ^14^, ^15^, ^16^

Therefore, our study aims to: (1) describe the distribution of caesarean delivery across individual‐level demographic, medical, socioeconomic and institutional factors overall and across 36 states/union territories; (2) assess the extent of variation in caesarean delivery attributable to three geographic/population levels (states, districts and communities); (3) estimate the proportion of variance in caesarean delivery at each population level explained by a set of 20 individual‐level demographic, socioeconomic, health/medical and institutional factors; and (4) finally examine aims 1–3 by type of delivery facilities (i.e. public vs. private).

## 2 METHODS

### 2.1 Data source, study design and study population

Data for this study were derived from the India NFHS‐4, collected in 2016.^5^ NFHS is a nationally representative survey that comprehensively provides information on indicators related to maternal and child health. NFHS‐4 adopted a stratified two‐stage sampling for both rural and urban areas. Villages and census enumeration blocks served as the primary sampling units (PSU) in rural and urban areas, respectively, and were selected with probability proportional to population size in the first stage, followed by a random selection of households within each PSU at the second stage.^5^

Our study sample was defined as women who had an institutional delivery for the most recent single live birth (excluding twins) during the 5 years preceding the survey. More detailed descriptions of data collection processes are provided in DHS technical documentation.^5^

### 2.2 Geographic level

Four levels of analysis were conceptualised and operationalised for the purpose of this study where the individual (within‐population) unit of inference was defined as child (or mother) (level 1) and three geographic (between‐population) units of inference included communities (level 2), districts (level 3) and states/union territories (level 4). Each level has political, administrative, social and cultural implications that could potentially affect the distribution of caesarean delivery. For example, in India, states (and union territories) are the highest administrative/political level at which federal health policies operate. Districts are the lowest administrative level at which the elected district councils plan the provision of diverse resources, services and infrastructures for health. Lastly, communities represent women's and children's local environments, which is generally villages for rural areas and survey blocks for urban areas.

### 2.3 Outcome

The outcome was caesarean delivery, which was derived from the ‘woman's questionnaire’ pertaining to the question ‘Was (NAME) delivered by cesarean section, that is, did they cut your belly open to take the baby out?’ Caesarean delivery was coded as yes or no.

### 2.4 Exposure

In order to examine the extent to which variation in caesarean delivery was accounted for by individual characteristics, we identified a set of 20 covariates based on literature review and theoretical frameworks. Sociodemographic characteristics included mother's age at childbirth, birth order, baby gender, religion, caste, type of residence (urban or rural), maternal education, household wealth quintile and paternal education. Health and medical factors included low birthweight, mother's perception of baby size at birth, maternal height, BMI, smoking, chewing tobacco, drinking alcohol, and history of miscarriage, abortion or stillbirth. Institutional factors included insurance coverage and antenatal care, and the type of delivery facility. The type of facility was categorised into public and private. Public facilities included the following: government or municipality hospital, government dispensary, urban health centre or post, an urban family welfare centre, community health centre, block primary health centre and subcentre. Private facilities included the following: private hospital, maternity home, and clinic, non‐government organisation and trust hospital and clinic. The categorisation and unit of each exposure variable can be referred to Table S2.

### 2.5 Statistical analysis

We estimated unadjusted rates of caesarean delivery for the country of India and by state and district. Then, we presented ‘adjusted’ distributions of caesarean delivery for India, which consider all geographic levels simultaneously and quantified the extent to which accounting for clustering of individual factors explains geographic variation in caesarean delivery in each level. While these analyses are explicitly focused on examining variation (random effects) in caesarean delivery, we present measures of association (fixed effects) for each of the 20 individual factors included in adjusted models.

A series of four‐level random intercept logistic regression model was utilised to acquire variance estimates for each geographic level (state, district and communities) while simultaneously accounting for each of the other level. In our data set, respondents within the same community, which served as a PSU in the sampling of NFHS, are likely to be more alike than respondents of different communities. Our four‐level model addresses this clustering problem arising from survey sampling design by including community random effects.^20^, ^21^ Multilevel modelling was performed via Markov Chain Monte Carlo estimation procedures using a Gibbs sampler with default prior distributions of iterative generalised least squares estimation as starting values, a burn‐in of 500 cycles and monitoring of 5000 iterations of chains. We checked the chains of the loading estimates for all parameters for convergence.

Multilevel logistic regression models were specified according to the following general structure:

$\text{logit}\left(\mathrm{Pr}\;(Y_{\mathrm{ijkl}}=1\;|\;X)\right)=\beta_{0}+\beta X_{\mathrm{ijkl}}+\left(u_{0jkl}+v_{0kl}+f_{0l}\right)$, where the dependent variable Y (caesarean delivery) and independent variable X (representing a vector of covariates) were each assumed to follow a multilevel data structure whereby child *i* (level 1) is nested within community *j* (level 2), district *k* (level 3) and state *l* (level 4), with both fixed‐effects (*β*_0_, *βX_ijkl_*) and random‐effects parameters (*u*_0jkl_ at community level, *v_0kl_* at district level and *f_0l_* at state level). The random‐effects parameters are each assumed to follow a normal distribution with mean 0 and variances of $u_{0jkl}∼N\left(0,\sigma_{u_{0}}^{2}\right)$, $v_{0kl}∼N\left(0,\sigma_{v_{0}}^{2}\right)$ and $f_{0l}∼N\left(0,\sigma_{f_{0}}^{2}\right)$, respectively.^21^, ^22^, ^23^ Since logistic regression models do not have a level 1 residual term, the mother/child (level 1) variance was estimated as π^2^/3 (3.29) based on the method summarised by Goldstein et al.^24^

Two model specifications were estimated based on the general modelling structure outlined above: a null/unadjusted model (M0), which included only an intercept term in the fixed part of the model, and a fully adjusted model (M1). In order to develop a more detailed quantification of geographic variability in caesarean delivery, which is our study aim 2, we calculated the proportion of total *geographic* variance attributable to each population level; that is variance partitioning coefficient (VPC) calculated as the variance at relevant level divided by the ‘total geographic variance’ in M0 and M1, respectively. Further, the proportion of between‐population variance explained by the inclusion of the 20 individual characteristics in M1 compared with M0; that is proportional change in variance (PCV), was calculated at each level, which is our study aim 3.^23^ Technical details for VPC and PCV are included in the Supplementary Material.

We additionally performed stratified analyses by the type of delivery facility for all these models to examine the difference in VPCs and PCVs between public and private facilities, which is our study aim 4. Lastly, maps were produced to visualize the geographic distribution of caesarean delivery. First, we mapped mean caesarean delivery across 640 districts to visualize unadjusted variation in caesarean use at the district level. Second, we visualized the extent of within‐district variation in caesarean delivery by mapping the standard deviations of village‐specific residuals within each district. These were estimated by extracting 27,218 village‐specific residuals ($u_{0jkl}$) from the fully adjusted model and calculating the standard deviation for each of their corresponding 640 districts. Maps were produced for the entire sample and by type of delivery facility. All models were estimated using MLwiN 3.0 software program.^25^ Maps were produced in ArcGIS Pro (version 2.0), and figures, calculations of variance summary metrics, and residuals analyses were performed using the R programming language (version 4).

### 2.6 Missing data

Of 146,713 observations of women who had an institutional delivery for their most recent single‐child birth during the 5 years preceding the survey, 4.8% in caste, 1.3% in BMI and 1.3% in maternal height were missing. The main results were based on complete case analyses of 136,985 observations. Since a total 6.6% of the sample were missing, we conducted a sensitivity analysis replicating the methods detailed above utilising multiple imputation. The multiple imputation analysis was based on m = 20 imputations and an imputation model that included all dependent and independent variables in the fully adjusted (m1) model.

### 2.7 Ethics approval

Ethics approval from our respective institutions was not required because our study was limited to the publicly available NFHS‐4 data set that contained no personally identifiable information.

## 3 RESULTS

### 3.1 Sample characteristics and prevalence of caesarean delivery

The final analytic sample was composed of 136,985 births nested hierarchically within 27,218 communities, 640 districts and 36 states (Table S1). Of these, 19.3% (*n* = 26,446) were born via caesarean delivery. Most births took place in public facilities (*n* = 97,465; 71.2%) rather than private facilities (*n* = 39,520; 28.8%). However, caesarean deliveries occurred more frequently in private (*n* = 15,601; 39.5%) vs. public facilities (*n* = 10,845; 11.1%) (*p* < .001) (Table 1).

**TABLE 1:** Individual characteristics and caesarean delivery percentages for Indian women in the NFHS‐4 (2016), overall and by the type of delivery facility

Variable	Overall		Public		Private	
	Total births	Caesarean delivery *N* (%)	Total births	Caesarean delivery *N* (%)	Total births	Caesarean delivery *N* (%)
Total	136,985	26,446 (19.3)	97,465	10,845 (11.1)	39,520	15,601 (39.5)
Demographic factors						
Mother's age at childbirth (years)						
<20	4351	659 (15.1)	7784	764 (9.8)	2475	875 (35.4)
20–29	94,172	17,446 (18.5)	70,192	7638 (10.9)	29,197	11,285 (38.7)
30–34	25,859	5692 (22.0)	11,721	1524 (13.0)	5609	2401 (42.8)
≥35	12,603	2649 (21.0)	4666	555 (11.9)	1983	895 (45.1)
Birth order						
First	50,939	12,943 (25.4)	32,296	4951 (15.3)	16,867	7593 (45.0)
Second	47,060	9780 (20.8)	32,281	4026 (12.5)	13,957	5649 (40.5)
Third	21,552	2614 (12.1)	16,046	1041 (6.5)	5214	1581 (30.3)
Four or more	17,434	1109 (6.4)	13,740	463 (3.4)	3226	633 (19.6)
Baby gender						
Male	62,128	12,046 (19.4)	43,185	4894 (11.3)	17,462	6925 (39.7)
Female	74,857	14,400 (19.2)	51,178	5587 (10.9)	21,802	8531 (39.1)
Religion						
Hindu	106,420	20,236 (19.0)	73,799	7745 (10.5)	30,110	12,125 (40.3)
Muslim	15,586	3292 (20.8)	9940	1318 (13.3)	5441	1878 (34.5)
Christian	8727	1531 (17.5)	6619	794 (12.0)	1845	697 (37.8)
Other	5982	1387 (23.2)	4005	624 (15.6)	1868	756 (40.5)
Socioeconomic factors						
Maternal education						
No education	31,117	2766 (8.9)	24,896	1246 (5.0)	5146	1418 (27.6)
Primary graduate or less	17,679	2291 (13.0)	13,744	1119 (8.1)	3375	1108 (32.8)
Secondary graduate or less	70,228	14,953 (21.3)	48,273	6546 (13.6)	20,444	8100 (39.6)
College or above	17,961	6436 (35.8)	7450	1570 (21.1)	10,299	4830 (46.9)
Type of residence						
Urban	38,872	11,052 (28.4)	21,518	3798 (17.7)	16,611	7102 (42.8)
Rural	98,113	15,394(15.7)	72,845	6683 (9.2)	22,653	8354 (36.9)
Caste						
Scheduled caste	26,844	4531 (16.9)	20,555	2244 (10.9)	5523	2170 (39.3)
Scheduled tribe	23,565	2975 (11.2)	19,489	1690 (8.7)	3358	1187 (35.3)
Other backward class	58,461	11,371 (19.5)	38,075	3946 (10.4)	19,007	7237 (38.1)
Others	28,115	7569 (26.9)	16,244	2601 (16.0)	11,376	4862 (42.7)
Wealth level						
1st quintile (poorest)	25,922	1815 (7.0)	22,203	923 (4.2)	2906	812 (27.9)
2nd quintile	29,082	3389 (11.7)	23,521	1825 (7.8)	4671	1463 (31.3)
3rd quintile	29,232	5576 (19.1)	21,322	2731 (12.8)	7131	2708 (38.0)
4th quintile	27,460	7165 (26.1)	16,718	2833 (16.9)	10,177	4201 (41.3)
5th quintile (richest)	25,289	8501 (33.6)	10,599	2169 (20.5)	14,379	6272 (43.6)
Paternal education						
No education	3006	298 (9.9)	2415	133 (5.5)	466	151 (32.4)
Primary graduate or less	2991	397 (13.3)	2341	196 (8.4)	545	192 (35.2)
Secondary graduate or less	13,947	2946 (21.1)	9630	1330 (13.8)	4002	1551 (38.8)
College or above	4032	1279 (31.7)	1834	301 (16.4)	2149	963 (44.8)
No paternal survey	113,009	21,526 (19.0)	81,245	8885(11.0)	33,544	12,744 (38)

Low birthweight (<2500 grams)						
Yes	20,636	4257 (20.0)	14,169	1726 (12.2)	6085	2580 (42.4)
No	106,245	21,247 (20.0)	73,412	8379 (11.4)	30,566	12,415 (40.6)
Not weighed at birth/don't know	10,104	942 (9.3)	6782	376 (5.5)	2613	461 (17.6)
Baby size^a^						
Very large	7700	1903 (24.7)	5027	750 (14.9)	2454	1113 (45.4)
Larger than average	17,981	4065 (22.6)	12,014	1618 (13.5)	5413	2353 (43.5)
Average	95,810	17,613 (18.4)	66,701	6964 (10.4)	26,896	10,267 (38.2)
Smaller than average	11,900	2188 (18.4)	8299	909 (11.0)	3368	1282 (38.1)
Very small	3594	677 (18.8)	2322	240 (10.3)	1133	441 (38.9)
Mother's height (cm)						
<145	14,338	2911 (20.3)	10,475	1271 (12.1)	3371	1556 (46.2)
145 −149	35,195	6547 (18.6)	25,382	2723 (10.7)	8865	3662 (41.3)
150 −154	47,099	8766 (18.6)	32,507	3410 (10.5)	13,449	5191 (38.6)
155 −159	28,882	5759 (20.0)	19,010	2178 (11.5)	9344	3506 (37.5)
≥160	11,471	2463 (21.4)	6989	899 (12.9)	4235	1541 (36.4)
BMI (kg/m^2^)						
<16.0	4252	513 (12.1)	2050	671 (32.7)	2479	1429 (57.6)
16.0 −18.4	25,880	3049 (11.8)	9851	2154 (21.9)	7705	3792 (49.2)
18.5 −24.9	84,195	14,700 (17.5)	59,348	6100 (10.3)	22,725	8298 (36.5)
25.0 −29.9	17,913	6027 (33.6)	19,898	1330 (6.7)	5419	1664 (30.7)
≥30.0	4745	2175 (45.5)	3216	226 (7.0)	936	273 (29.2)
Smoking						
Yes	1318	177 (13.4)	9314	876 (9.4)	1861	642 (34.5)
No	135,667	26,269 (19.4)	85,049	9605 (11.3)	37,403	14,814 (39.6)
Chewing tobacco						
Yes	8915	1306 (14.6)	7045	735 (10.4)	1574	538 (34.2)
No	128,070	25,140 (19.6)	87,318	9746 (11.2)	37,690	14,918 (39.6)
Drinking alcohol						
Yes	2187	336 (15.4)	1768	168 (9.5)	340	154 (45.3)
No	134,798	25,615(19.4)	92,595	10,313 (11.1)	38,924	15,302 (39.3)
Miscarriage, abortion or stillbirth						
No	14,891	3510 (23.6)	9337	1258 (13.5)	5166	2182 (42.2)
Yes	122,094	22,936 (18.8)	85,026	9223 (10.8)	34,098	13,274 (38.9)
Institutional factors						
Insurance						
Covered	20,958	4690 (22.4)	14,877	1930 (13.0)	5579	2670 (47.9)
Not covered	116,027	21,756 (18.8)	79,486	8551 (10.8)	33,685	12,786 (38.0)
Antenatal care more than 4 times						
Yes	73,403	18,546 (25.3)	47,092	7323 (15.6)	25,611	11,144 (43.5)
No	63,582	7900 (12.4)	47,271	3158 (6.7)	13,653	4312 (31.6)

^a^ Mother's perception of baby size at birth.

### 3.2 Geographic variation in caesarean delivery prevalence

**FIGURE 1:**
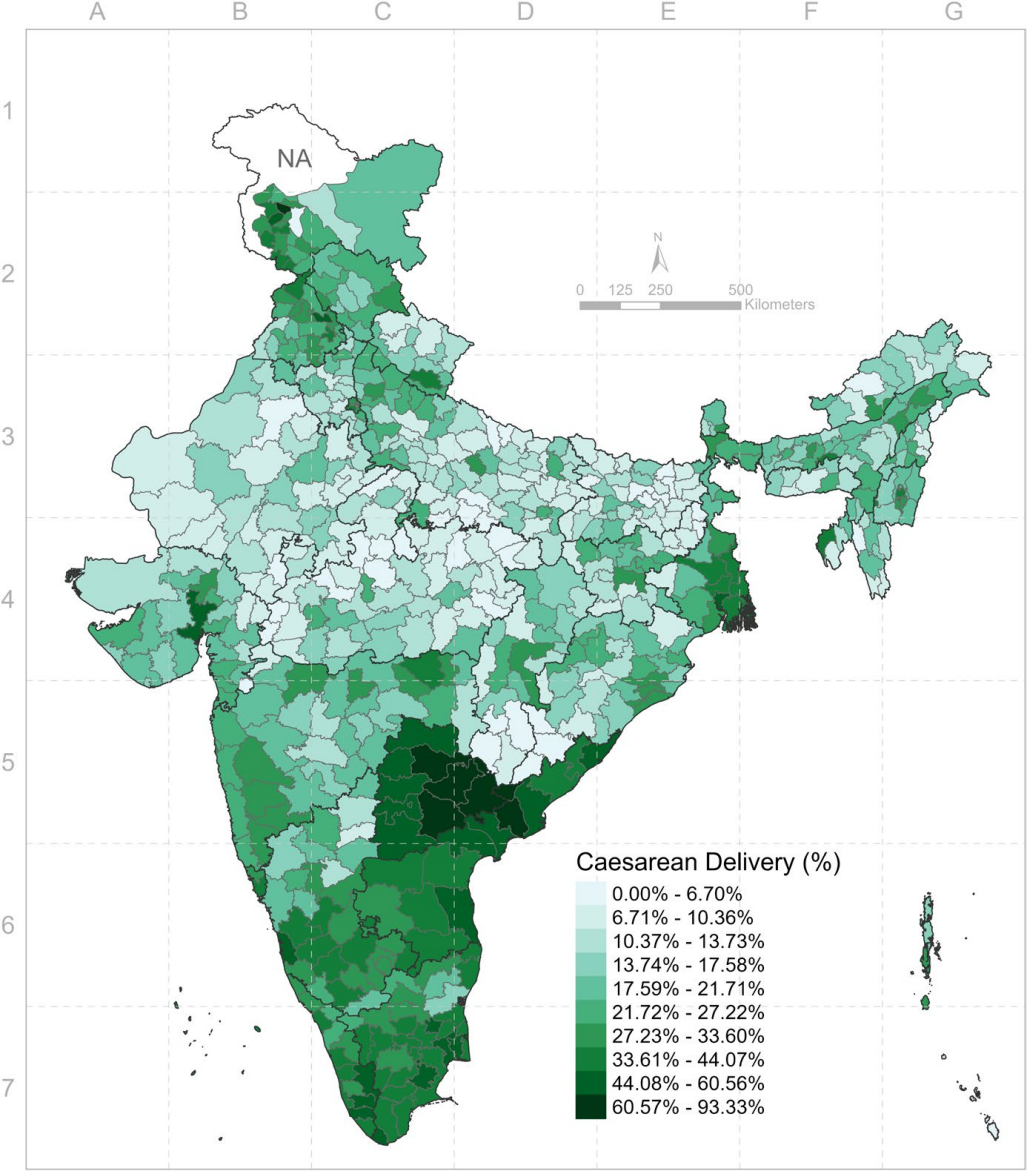
Distribution of caesarean delivery prevalence across 640 districts of India in 2016 [Colour figure can be viewed at wileyonlinelibrary.com]

The lowest prevalence of caesarean delivery was found in Bihar (10.7%) (Table 2). However, the gap between public and private facilities was large (2.8% in public vs. 33.3% in private). On the other hand, Telangana was the highest in caesarean delivery rate (62.1%) and also with a large difference between public (39.7%) and private facilities (74.8%). District‐level rate of caesarean delivery also varied considerably from a minimum of 0% to a maximum of 93.3% (Figure 1).

**TABLE 2:** State‐specific caesarean delivery percentages in India in 2016, overall and by the type of delivery facility

State^a^	Overall		Public		Private	
	Total births (%)	Caesarean (%)^b^	Total births (%)	Caesarean (%)^b^	Total births (%)	Caesarean (%)^b^
Total	136,985 (100)	19.3	97,465 (100)	11.1	39,520 (100)	39.5
Telangana	1483 (1.1)	62.1	537 (0. 6)	39.7	946 (2.4)	74.8
Andhra Pradesh	1961 (1.4)	44.6	843 (0.9)	26.0	1118 (2.8)	58.6
Puducherry	862 (0.6)	42.0	537 (0.6)	33.5	325 (0.8)	56.0
Lakshadweep	249 (0.2)	38.6	160 (0.2)	28.8	89 (0.2)	56.2
Tamil Nadu	5986 (4.4)	36.5	3986 (4.1)	27.9	2000 (5.1)	53.6
Kerala	1964 (1.4)	35.2	810 (0.8)	31.7	1154 (2.9)	37.6
Goa	288 (0.2)	34.7	167 (0.2)	24.6	121 (0.3)	48.8
Manipur	2755 (2.0)	30.1	1859 (1.9)	21.6	896 (2.3)	47.7
Delhi	900 (0.7)	29.8	602 (0.6)	22.4	298 (0.8)	44.6
West Bengal	2748 (2.0)	28.6	2123 (2.2)	16.6	625 (1.6)	69.6
Jammu and Kashmir	2850 (2.1)	28.6	2677 (2.7)	26.6	173 (0.4)	59.5
Punjab	3743 (2.7)	26.8	2178 (2.2)	18.3	1565 (4.0)	38.7
Karnataka	4820 (3.5)	25.9	3244 (3.3)	17.0	1576 (4.0)	44.0
Chandigarh	129 (0.1)	24.8	97 (0.1)	18.6	32 (0.1)	43.8
Daman and Diu	259 (0.2)	24.3	115 (0.1)	10.4	144 (0.4)	35.4
Tripura	752 (0.5)	23.0	652 (0.7)	15.6	100 (0.3)	71.0
Himachal Pradesh	1671 (1.2)	22.3	1341 (1.4)	16.4	330 (0.8)	46.4
Maharashtra	6055 (4.4)	21.8	3421 (3.5)	13.1	2634 (6.7)	33.1
Sikkim	820 (0.6)	21.5	736 (0.8)	17.7	84 (0.2)	54.8
Assam	4830 (3.5)	21.4	4084 (4.2)	15.2	746 (1.9)	55.5
Uttarakhand	2844 (2.1)	20.0	1953 (2.0)	10.2	891 (2.3)	41.4
Dadra and Nagar Haveli	210 (0.2)	20.0	155 (0.2)	12.9	55 (0.1)	40.0
Gujarat	4749 (3.5)	19.3	1881 (1.9)	9.3	2868 (7.3)	25.9
Nagaland	983 (0.7)	18.2	780 (0.8)	13.5	203 (0.5)	36.5
Odisha	7353 (5.4)	17.0	6514 (6.7)	11.8	839 (2.1)	57.0
Jharkhand	5568 (4.1)	16.4	3834 (3.9)	5.0	1734 (4.4)	41.6
Arunachal Pradesh	1790 (1.3)	15.9	1542 (1.6)	12.1	248 (0.6)	39.9
Uttar Pradesh	19,535 (14.3)	15.3	12,374 (12.7)	4.9	7161 (18.1)	33.2
Meghalaya	1658 (1.2)	15.1	1303 (1.3)	10.0	355 (0.9)	34.1
Andaman and Nicobar Islands	470 (0.3)	15.1	459 (0.5)	13.5	11 (0.0)	81.8
Haryana	4671 (3.4)	14.8	2998 (3.1)	9.5	1673 (4.2)	24.2
Chhattisgarh	4784 (3.5)	14.5	3806 (3.9)	5.8	978 (2.5)	48.4
Mizoram	2825 (2.1)	13.0	2495 (2.6)	10.3	330 (0.8)	33.0
Madhya Pradesh	13,446 (9.8)	11.7	11,479 (11.8)	6.3	1967 (5.0)	43.3
Rajasthan	9897 (7.2)	11.4	7509 (7.7)	6.8	2388 (6.0)	25.9
Bihar	11,077 (8.1)	10.7	8214 (8.4)	2.8	2863 (7.2)	33.3

^a^ States were rank‐ordered from the highest to the lowest based on overall caesarean delivery rates.

^b^ Caesarean %: caesarean delivery percentage.

### 3.3 Variance decomposition

**FIGURE 2:**
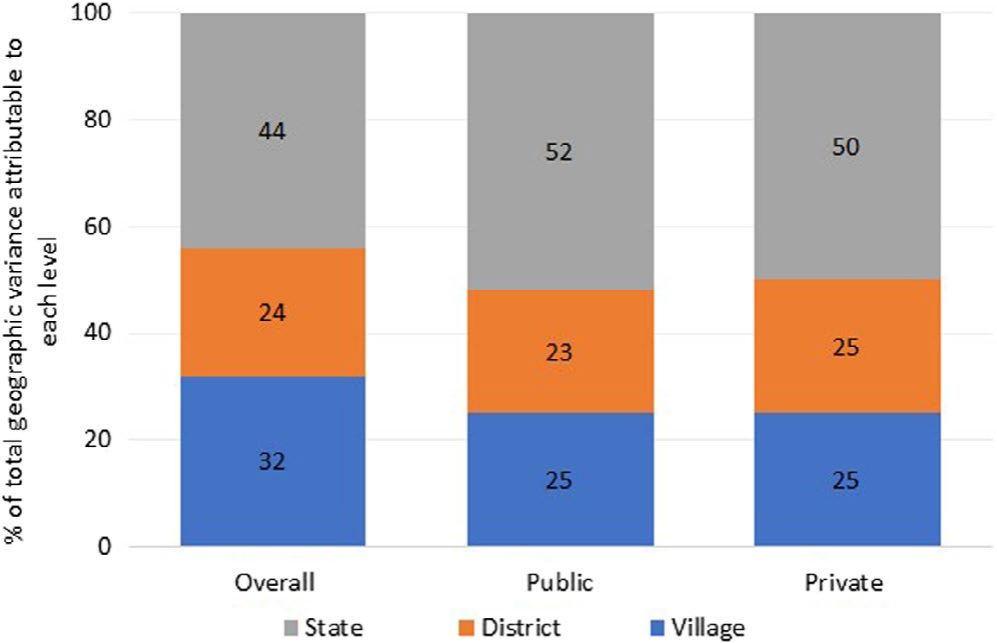
Partitioning geographic variation in caesarean delivery among 136,985 women in India, overall and by the type of delivery facility

Variance estimates from unadjusted and adjusted random intercept models are presented in Table 3. Total variance in caesarean delivery estimated from the unadjusted model (M0) was 4.21 (0.41 at state level, 0.21 at district level, 0.30 at community level and π^2^/3 = 3.29 at individual level). Removing the constant (π^2^/3) yields a total geographic variance of 0.92, which takes into account 22% of the total variance (0.92/4.21). Of the total geographic variance, 44% was attributable to states, 24% to districts and 32% to communities (Figure 2). Results from stratified analyses by the type of delivery facilities showed a greater total geographic variance in caesarean delivery among public facilities than among private facilities (Table 3). For example, a total geographic variance was 1.11 and 0.67 before and after adjustment, respectively, for the public facility, while it was 0.64 and 0.50, respectively, for the private facility. Of the total geographic variance in caesarean delivery in public facilities, 52%, 23% and 25% were attributable to states, districts and communities, respectively. Similarly, of the total geographic variance in private facilities, 50%, 25% and 25% were attributable to states, districts and communities, respectively (Figure 2).

**TABLE 3:** Population variance in caesarean delivery and variance explained among 136,985 women in India in 2016, comparing null (M0) to fully adjusted (M1) models, overall and by the type of delivery facility

Group level	Overall		Public		Private	
	M0	M1	M0	M1	M0	M1
State						
Variance estimate (SE)	0.41 (0.11)	0.23 (0.08)	0.57 (0.08)	0.32 (0.06)	0.32 (0.07)	0.25 (0.07)
Variance explained (%)	—	44	—	44	—	20
District						
Variance estimate (SE)	0.21 (0.02)	0.10 (0.01)	0.26 (0.07)	0.16 (0.05)	0.16 (0.06)	0.13 (0.05)
Variance explained (%)	—	52	—	38	—	19
Community						
Variance estimate (SE)	0.30 (0.003)	0.16 (0.002)	0.28 (0.07)	0.19 (0.05)	0.16 (0.06)	0.12 (0.05)
Variance explained (%)	—	46	—	30	—	24

Note

M0: null model/M1: adjusted for all individual characteristics (20 for overall, 19 for public and private).

Abbreviation: SE, standard error.

### 3.4 Proportion in variation attributable to clustering of individual‐level risk factors

**FIGURE 3:**
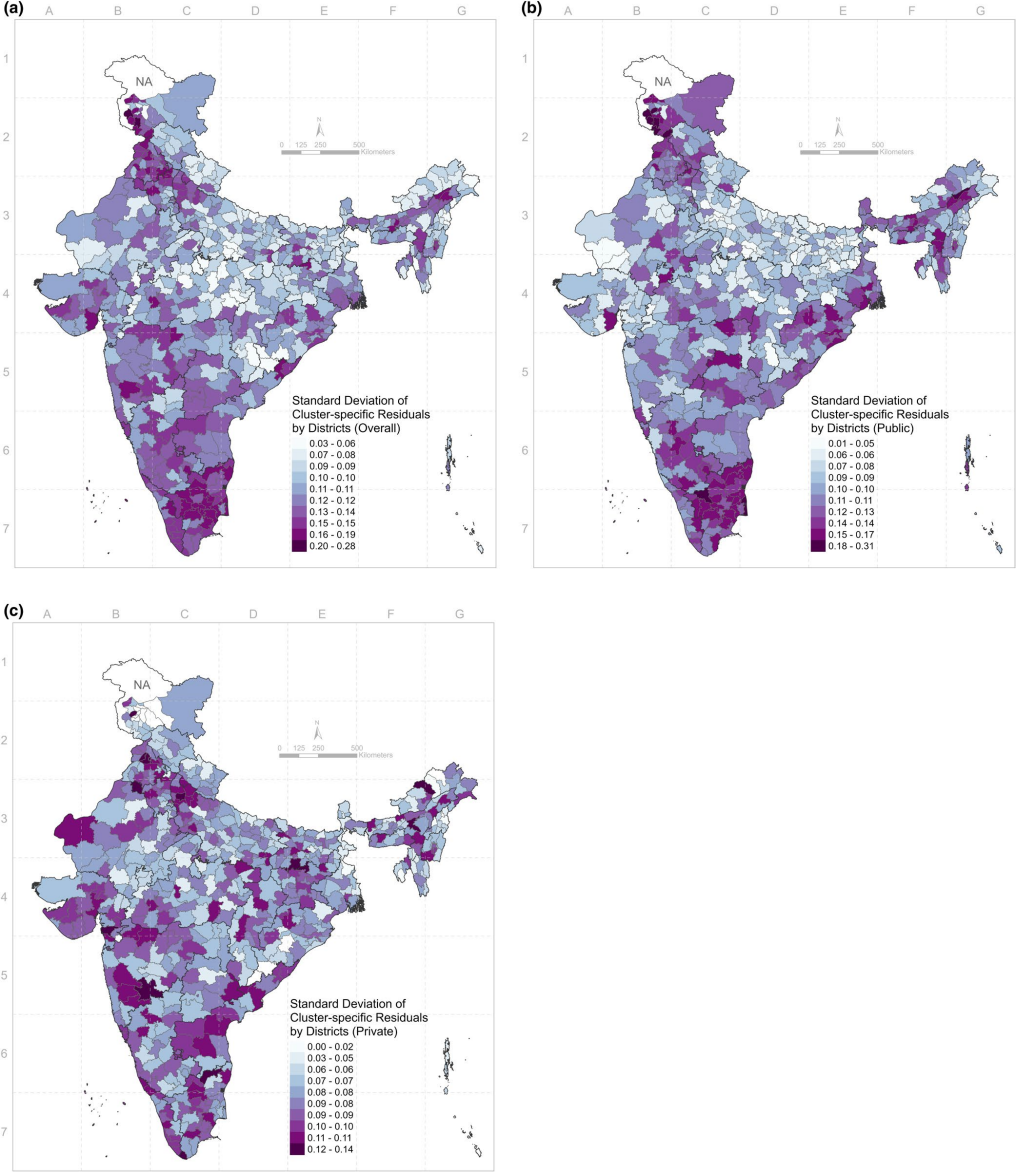
Distribution of standard deviation of cluster‐specific residuals by 640 districts: overall (A), public (B) and private (C) [Colour figure can be viewed at wileyonlinelibrary.com]

Of the total geographic variance in caesarean delivery, 44% of the between‐state variance, 52% of the between‐district variance and 46% of the between‐community variance were explained away by the addition of 20 individual‐level covariates (Table 3). Of the between‐state variance in caesarean delivery in public facilities, 44% was explained by the 19 individual‐level factors (excluding the type of facility from 20 covariates), whereas only 20% of the between‐state variance was accounted for in private facilities. Similarly, adjusting for the 19 individual characteristics explained 38% of the between‐district variance and 30% of the between‐community variance in public facilities, but only 19% and 24%, respectively, in private facilities. Even though the proportion of variance explained by a set of individual‐level risk factors was larger in public facilities, the remaining variation after adjustment was still larger for the public than for the private facilities (Table 3). After adjusting for all the individual‐level risk factors, substantial variation remained between‐clusters within‐district. In maps visualising the post‐adjustment standard deviation of cluster‐specific residuals in caesarean delivery by districts (Figure 3A‐C), larger variation was observed within districts in Southern India. The range of standard deviation of cluster‐specific residuals was greater for public facilities compared to private facilities, indicating that the remaining unexplained variation in caesarean delivery at the community level was larger in the public facility.

### 3.5 Sensitivity analyses

Results from multiple imputation analyses are provided in Tables S3 and S4 and Figure S1. Both fixed‐effects and random‐effects estimates were nearly identical for M0 and M1 in imputed and original results.

## 4 COMMENT

### 4.1 Principal findings

The average rate of caesarean delivery in India (19.3%) obscures considerable within‐country variation in caesarean delivery. State‐specific prevalence varied from a low of 10.7% in Bihar to a high of 62.1% in Telangana. From the multilevel analysis, more than 20% of the total variation in caesarean delivery was estimated as between‐population geographic variability. Of the total geographic variation in caesarean delivery, 44% was attributable to states, followed by communities (32%), and lastly districts (24%). Public facilities showed greater geographic variation in caesarean delivery than private facilities, with total geographic variance estimates of 1.11 and 0.64, respectively. Upon fully accounting for the complete set of 19 individual risk factors, reductions in variance terms were larger for public facilities at all three geographic levels than for private facilities. However, the remaining variation was still greater among public vs. private facilities at all three levels.

### 4.2 Strengths of the study

Our study is the first to demonstrate that each geographic level substantially contributes to the shaping of caesarean delivery distribution in India and therefore indicating that single‐level studies may provide an incomplete and sometimes misleading understanding of the distribution of caesarean delivery. We also demonstrated that a substantial proportion of between‐population variation still existed even after clustering of individual risk factors was comprehensively adjusted for, indicating that contextual factors may contribute to generating between‐population variation at the macro‐ and micro‐geographic unit.

### 4.3 Limitations of the data

Although our study has many conceptual and methodological strengths, it also includes a few limitations. First, our study variables including caesarean delivery are based on self‐report. Although experiencing a surgical procedure is unlikely to be something that someone would forget or incorrectly recount, there are chances of recall bias for other self‐report variables (eg baby size at birth). However, measurement error associated with these independent variables is likely to be random, such that adjusted models yield conservative estimates of percent explained—which is of greater conceptual importance to the objective of this study—compared with precise fixed‐effects estimates. Second, although the multilevel data structure of the present study reflects sampling design of the NFHS‐4 data and the contextual reality of multiple units of administrative, geographic and political significance in India, even our four‐level model may be at risk of the missing unit problem. For example, subnational administrative units such as division (higher than the district and lower than state), subdistrict (lower than district but higher than community) or geographic units such as zone or region may also have factors shaping the between‐population variation in caesarean delivery. Finally, although we tried to control for individual risk factors for caesarean delivery as comprehensively as the NFHS data allowed, it is likely that there are still other important factors missing in NFHS data such as medical factors indicating emergency situation for caesarean surgery.

### 4.4 Interpretation

The findings presented herein are highly relevant to the current policy discussions regarding caesarean delivery in India and globally. Prevalence of caesarean delivery at the district and state level was considerably heterogeneous, indicating that the overall rate masks the variation across multiple geographic levels within India.

Results from unadjusted and adjusted multilevel models demonstrate that differing contexts respond differently to individual determinants of caesarean delivery. For example, despite between‐state differences accounting for the greatest proportion of overall variation in caesarean delivery, they were the least amenable to explanation via individual characteristics, while districts accounted for the least amount of total variation but were most explainable by the aforementioned risk factors. A substantial level of variation in caesarean delivery still remained unexplained even after full adjustment of all individual risks in all levels, indicating that structural attributes missing in our model would likely shape between‐population variation in caesarean delivery. For example, at the community level, different social norms and cultures formulated within a community, different forms and quality of community health workers' activities, or geographic accessibility to the facilities having surgical capacity would affect the caesarean delivery rate within a community. At the district or state level, health policies such as payment to providers or service delivery mechanisms could affect the caesarean delivery.

The proportion of the variation in caesarean delivery explained by the individual characteristics was smaller in the private facility, indicating that structural factors drive geographic variation in caesarean delivery more strongly among the private facility than among the public facility. The public‐private partnership (PPP) programmes performed as a state initiative, for example the *Chiranjeevi Yojana* in Gujarat or *Ayushmati Scheme* in West Bengal, can be one example of those structural factors.^26^, ^27^, ^28^ As mentioned previously, the state only funds services provided by public providers in India. However, through the PPP, the state pays private obstetricians to tackle the problem of the lack of obstetricians in public facilities so that pregnant mothers can give birth at private facilities without concerns about the cost. However, due to higher reimbursement to providers for caesarean delivery than vaginal delivery, caesarean delivery disproportionally increased among private facilities during the programme, which led some states to modify the programme to pay a fixed sum for a specific unit of deliveries regardless of the type of the deliveries, acting as an embedded disincentive for unnecessary caesarean delivery.^26^, ^27^, ^28^ This led to a decrease again in caesarean delivery. These state‐level policies may shape the between‐state variation in caesarean delivery.

The high degree of variation in caesarean delivery presented in this study suggests that rates in some locales may be too high and too low in others. That is, high caesarean prevalence observed in some states may be to some degree composed of medically unnecessary caesarean deliveries, which may not only adversely affect health outcomes for individual mothers and babies, but also occupy resources that could have been utilised elsewhere with possibly greater medical need. On the other hand, low caesarean prevalence observed in other states may indicate unmet need for medically necessary caesarean deliveries, thus contributing to avoidable morbidity and mortality. Therefore, it may be useful to identify optimal caesarean delivery rates appropriate for the context of multiple geographic levels, and monitor them appropriately.

## 5 CONCLUSIONS

Our study results highlight the importance of understanding geographic variation at multiple levels as illustrated through a comprehensive accounting of the variations in caesarean delivery in India. Tailored contextual interventions may reduce between‐population variations in caesarean access and utilisation, beyond those targeting individual characteristics alone. Specific policy recommendations are beyond the scope of this paper. Future studies should investigate the potential of specific interventions by identifying more diverse contextual factors driving between‐population variations. Such studies will require more extensive data including detailed clinical, administrative and sociocultural information.

## CONFLICT OF INTEREST

All authors declare no conflict of interest.

## 6 ACKNOWLEDGEMENTS

None.

## Supporting information

Supplementary Material

## Data Availability

The data that support the findings of this study are available in the Demographic and Health Survey program at https://dhsprogram.com/data/dataset/India_Standard‐DHS_2015.cfm?flag=0
