# Supplementary material for: Geographic variation in caesarean delivery in India
Source: Paediatr Perinat Epidemiol. 2021 Aug 31;36(1):92–103. doi: 10.1111/ppe.12807 (PMC9292746; doi:10.1111/ppe.12807)
Supplement: Supplementary file 1 — Supplementary Material [file PPE-36-92-s001.docx]

**Full Title**

Geographic Variation in Caesarean Delivery in India.

**Study Authors**

Justin Rodgers, Hwa-Young Lee, Rockli Kim, Nachiket Mor, SV Subramanian

**Supplemental Materials**

List of Supplementary Tables and Figures:

Text S1. Technical details for variance partition coefficient (VPC) and proportional change in variance (PCV) calculations

Table S1. Distributions of districts, communities, and individuals for each of the 36 states/union territories in India in 2016

Table S2. Associations between individual risk factors and caesarean delivery among 136,985 women in India in 2016, overall and by the type of delivery facility.

Table S3. Results from multiple imputation analysis: associations between individual risk factors and caesarean delivery among 146,713 women in India in 2016, overall and by the type of delivery facility.

Table S4. Results from multiple imputation analysis: Population variance in cesarean delivery and variance explained among 146,713 women in India in 2016 comparing null (M0) to fully adjusted (M1) models, overall and by the type of delivery facility.

Figure S1. Results from multiple imputation analysis: Partitioning geographic variation in cesarean delivery among 146,713 women in India in 2016, overall and by the type of delivery facility.

Text S1. Technical details for variance partition coefficient (VPC) and proportional change in variance (PCV) calculations

1. The proportion of total geographic variance in cesarean delivery (VPC) attributable to:

States = ($\frac{\sigma_{f_{0}}^{2}}{\sigma_{u_{0}}^{2}+\sigma_{v_{0}}^{2}+\sigma_{f_{0}}^{2}})x100$,

Districts = ( $\frac{\sigma_{v_{0}}^{2}}{\sigma_{u_{0}}^{2}+\sigma_{v_{0}}^{2}+\sigma_{f_{0}}^{2}})x100,$

Communities = $(\frac{\sigma_{u_{0}}^{2}}{\sigma_{u_{0}}^{2}+\sigma_{v_{0}}^{2}+\sigma_{f_{0}}^{2}})x100$

1. Proportional Change in Variance (PCV):

At state level = $\frac{{[\sigma_{u_{0}}^{2}+\sigma_{v_{0}}^{2}+\sigma_{f_{0}}^{2}]}_{model 1}-{[\sigma_{u_{0}}^{2}+\sigma_{v_{0}}^{2}+\sigma_{f_{0}}^{2}]}_{model 2}}{{[\sigma_{u_{0}}^{2}+\sigma_{v_{0}}^{2}+\sigma_{f_{0}}^{2}]}_{model 1}})x100$ $(\frac{{\sigma_{f_{0}}^{2}}_{M0}-{\sigma_{f_{0}}^{2}}_{M1}}{{\sigma_{f_{0}}^{2}}_{M0}})x100$,$(\frac{{[\sigma_{e_{0}}^{2}]}_{model 1}-{[\sigma_{e_{0}}^{2}]}_{model 2}}{{[\sigma_{e_{0}}^{2}]}_{model 1}})x100$

At district level = $\frac{{[\sigma_{u_{0}}^{2}+\sigma_{v_{0}}^{2}+\sigma_{f_{0}}^{2}]}_{model 1}-{[\sigma_{u_{0}}^{2}+\sigma_{v_{0}}^{2}+\sigma_{f_{0}}^{2}]}_{model 2}}{{[\sigma_{u_{0}}^{2}+\sigma_{v_{0}}^{2}+\sigma_{f_{0}}^{2}]}_{model 1}})x100$ $(\frac{{\sigma_{v_{0}}^{2}}_{M0}-{\sigma_{v_{0}}^{2}}_{M1}}{{\sigma_{v_{0}}^{2}}_{M0}})x100$,

At community level = $\frac{{[\sigma_{u_{0}}^{2}+\sigma_{v_{0}}^{2}+\sigma_{f_{0}}^{2}]}_{model 1}-{[\sigma_{u_{0}}^{2}+\sigma_{v_{0}}^{2}+\sigma_{f_{0}}^{2}]}_{model 2}}{{[\sigma_{u_{0}}^{2}+\sigma_{v_{0}}^{2}+\sigma_{f_{0}}^{2}]}_{model 1}})x100$ $(\frac{{\sigma_{u0}^{2}}_{M0}-{\sigma_{u_{0}}^{2}}_{M1}}{{\sigma_{u_{0}}^{2}}_{M0}})x100$

Table S1. Distributions of districts, communities, and individuals for each of the 36 states/union territories in India in 2016

| **State** | **District** | **Community** | **Individual** |
| --- | --- | --- | --- |
| Total | 640 | 27,218 | 136,985 |
| Telangana | 10 | 367 | 1,461 |
| Andhra Pradesh | 13 | 477 | 1,935 |
| Pondicherry | 4 | 152 | 861 |
| Lakshadweep | 35 | 35 | 214 |
| Tamil Nadu | 32 | 1,208 | 5,885 |
| Kerala | 14 | 505 | 1,857 |
| Goa | 2 | 72 | 291 |
| Manipur | 9 | 526 | 2,746 |
| Delhi | 9 | 280 | 886 |
| Jammu & Kashmir | 22 | 552 | 2,783 |
| West Bengal | 19 | 663 | 2,712 |
| Punjab | 20 | 755 | 3,679 |
| Karnataka | 30 | 1,086 | 4,708 |
| Chandigarh | 1 | 33 | 127 |
| Daman & Diu | 2 | 75 | 257 |
| Tripura | 4 | 195 | 716 |
| Himachal Pradesh | 12 | 441 | 1,639 |
| Maharashtra | 35 | 1,305 | 5,970 |
| Sikkim | 4 | 205 | 781 |
| Assam | 27 | 1,009 | 4,684 |
| Uttarakhand | 13 | 698 | 2,793 |
| Dadra & Nagar haveli | 1 | 38 | 199 |
| Gujarat | 26 | 969 | 4,630 |
| Nagaland | 11 | 388 | 965 |
| Orissa(odesha) | 30 | 1,406 | 7,212 |
| Jharkhand | 24 | 1,210 | 5,522 |
| Andaman & Nicobar Islands | 3 | 112 | 459 |
| Arunachal Pradesh | 16 | 605 | 1,706 |
| Meghalaya | 7 | 311 | 1,578 |
| Uttar Pradesh | 71 | 3,591 | 18,946 |
| Haryana | 21 | 793 | 4,609 |
| Chhattisgarh | 18 | 932 | 4,677 |
| Mizoram | 8 | 517 | 2,751 |
| Madhya Pradesh | 50 | 2,372 | 12,903 |
| Rajasthan | 33 | 1,624 | 9,686 |
| Bihar | 38 | 1,665 | 10,781 |

Table S2. Associations between individual risk factors and caesarean delivery among 136,985 women in India in 2016, overall and by the type of delivery facility.

| **Variable** | **Overall** | **Public** | **Private** |
| --- | --- | --- | --- |
|  | **Logit (SE)** | **Logit (SE)** | **Logit (SE)** |
| Intercept | -2.49 (0.15) | -2.78 (0.2) | -0.41 (0.21) |
| ***Demographic factors*** |  | | |
| Age at pregnancy (ref=<20 yrs) |  |  |  |
| 20-29 | 0.26 (0.05) | 0.17 (0.06) | 0.35 (0.08) |
| 30-34 | 0.58 (0.05) | 0.58 (0.07) | 0.6 (0.08) |
| >=35 | 0.82 (0.06) | 0.8 (0.08) | 0.84 (0.09) |
| Birth order (ref= Four or more) |  |  |  |
| Third | -0.34 (0.02) | -0.37 (0.03) | -0.31 (0.03) |
| Second | -0.87 (0.03) | -0.97 (0.04) | -0.74 (0.04) |
| First | -1.38 (0.04) | -1.5 (0.06) | -1.22 (0.06) |
| Baby gender (ref=male) |  |  |  |
| Female | -0.02 (0.02) | 0.00 (0.02) | -0.03 (0.02) |
| Religion (ref=other) |  |  |  |
| Hindu | -0.03 (0.05) | -0.05 (0.06) | -0.01 (0.07) |
| Muslim | -0.05 (0.05) | 0.07 (0.07) | -0.13 (0.07) |
| Christian | -0.16 (0.07) | -0.07 (0.09) | -0.23 (0.1) |
| ***Socioeconomic factors*** |  |  |  |
| Maternal education (ref=none) |  |  |  |
| Primary graduate or less | 0.12 (0.03) | 0.15 (0.04) | 0.07 (0.05) |
| Secondary graduate or less | 0.17 (0.03) | 0.23 (0.04) | 0.06 (0.04) |
| College or above | 0.23 (0.04) | 0.38 (0.05) | 0.12 (0.05) |
| Type or residency (ref=rural) |  |  |  |
| Urban | 0.09 (0.02) | 0.22 (0.03) | -0.04 (0.03) |
| Caste (ref=scheduled tribe) |  |  |  |
| Scheduled caste | -0.21 (0.04) | -0.22 (0.05) | -0.1 (0.06) |
| Other backward class | -0.06 (0.02) | -0.03 (0.03) | -0.08 (0.04) |
| Others | 0.09 (0.03) | 0.14 (0.04) | 0.07 (0.04) |
| Wealth level (ref=1^st^ quintile (poorest)) |  |  |  |
| 2^nd^ quintile | 0.14 (0.03) | 0.14 (0.04) | -0.08 (0.06) |
| 3^rd^ quintile | 0.4 (0.03) | 0.39 (0.04) | 0.06 (0.06) |
| 4^th^ quintile | 0.52 (0.04) | 0.56 (0.05) | 0.12 (0.06) |
| 5^th^ quintile (richest) | 0.53 (0.04) | 0.67 (0.06) | 0.18 (0.06) |
| Paternal education (ref=none) |  |  |  |
| Primary graduate or less | 0.05 (0.09) | 0.14 (0.12) | 0.02 (0.15) |
| Secondary graduate or less | 0.12 (0.07) | 0.28 (0.1) | -0.05 (0.12) |
| College or above | 0.02 (0.08) | 0.18 (0.12) | -0.1 (0.12) |
| No paternal survey | 0.07 (0.07) | 0.19 (0.09) | -0.07 (0.11) |
| ***Health and medical factors*** |  |  |  |
| Low Birth Weight |  |  |  |
| Yes (<2,500 grams) | 0.13 (0.02) | 0.11 (0.03) | 0.13 (0.04) |
| Missing (Not recorded/Unknown) | -0.4 (0.04) | -0.16 (0.06) | -0.71 (0.06) |
| Baby size (ref=very small) |  |  |  |
| Smaller than average | -0.09 (0.06) | -0.04 (0.08) | -0.07 (0.08) |
| Average | -0.09 (0.05) | -0.06 (0.08) | -0.05 (0.07) |
| Larger than average | 0.06 (0.06) | 0.09 (0.08) | 0.07 (0.08) |
| Very large | 0.17 (0.06) | 0.17 (0.09) | 0.19 (0.09) |
| Mother’s height (ref=<145cm) |  |  |  |
| 145 -149 | -0.32 (0.03) | -0.32 (0.04) | -0.35 (0.05) |
| 150 -154 | -0.52 (0.03) | -0.52 (0.04) | -0.54 (0.04) |
| 155 -159 | -0.6 (0.03) | -0.58 (0.04) | -0.63 (0.05) |
| >=160 | -0.64 (0.04) | -0.55 (0.05) | -0.7 (0.05) |
| BMI (ref=<16 .0kg/m^2^) |  |  |  |
| 16.0 -18.4 | -0.01 (0.06) | 0.04 (0.08) | 0.01 (0.08) |
| 18.5 -24.9 | 0.23 (0.05) | 0.3 (0.07) | 0.2 (0.08) |
| 25.0 -29.9 | 0.73 (0.06) | 0.84 (0.08) | 0.67 (0.08) |
| >=30.0 | 1.12 (0.06) | 1.26 (0.09) | 1.03 (0.09) |
| Smoking (ref=no) |  |  |  |
| Yes | 0.3 (0.09) | 0.26 (0.12) | 0.56 (0.18) |
| Chewing Tobacco (ref=no) |  |  |  |
| Yes | -0.01 (0.04) | 0.01 (0.05) | -0.04 (0.07) |
| Alcohol (ref=no) |  |  |  |
| Yes | -0.18 (0.07) | -0.17 (0.09) | -0.14 (0.13) |
| Miscarriage, abortion or stillbirth (ref=no) |  |  |  |
| Yes | 0.14 (0.02) | 0.16 (0.03) | 0.12 (0.03) |
| ***Institutional factors*** |  | | |
| Insurance (ref=not covered) |  |  |  |
| Covered | 0.07 (0.02) | 0.05 (0.03) | 0.11 (0.04) |
| Place of delivery (ref=public) |  |  |  |
| Private | 1.48 (0.02) | - | - |
| ANC more than 4 times (ref=no) |  |  |  |
| Yes | 0.24 (0.02) | 0.22 (0.03) | 0.22 (0.03) |
| Notes: Fixed effects (logit) parameter estimates presented above derived from multilevel logistic regression models. M0 refers to intercept-only (null) model; M1 refers to models fully adjusted for sociodemographic + medical + institutional factors. | | | |

Table S3. Results from multiple imputation analysis: associations between individual risk factors and caesarean delivery among 146,713 women in India in 2016, overall and by the type of delivery facility.

| **Variable** | **Overall** | **Public** | **Private** |
| --- | --- | --- | --- |
|  | **Logit (SE)** | **Logit (SE)** | **Logit (SE)** |
| Intercept | -2.46 (0.15) | -2.79 (0.19) | -0.41 (0.21) |
| ***Demographic factors*** |  | | |
| Age at pregnancy (ref=<20 yrs) |  |  |  |
| 20-29 | 0.28 (0.05) | 0.17 (0.06) | 0.35 (0.08) |
| 30-34 | 0.61 (0.05) | 0.58 (0.07) | 0.59 (0.08) |
| >=35 | 0.83 (0.06) | 0.80 (0.08) | 0.84 (0.09) |
| Birth order (ref= Four or more) |  |  |  |
| Third | -0.34 (0.02) | -0.37 (0.03) | -0.31 (0.03) |
| Second | -0.87 (0.03) | -0.97 (0.04) | -0.74 (0.04) |
| First | -1.38 (0.04) | -1.50 (0.06) | -1.22 (0.06) |
| Baby gender (ref=male) |  |  |  |
| Female | -0.01 (0.02) | 0.00 (0.02) | -0.02 (0.02) |
| Religion (ref=other) |  |  |  |
| Hindu | -0.06 (0.04) | -0.05 (0.06) | -0.01 (0.07) |
| Muslim | -0.09 (0.05) | 0.07 (0.07) | -0.13 (0.07) |
| Christian | -0.16 (0.06) | -0.07 (0.09) | -0.23 (0.10) |
| ***Socioeconomic factors*** |  |  |  |
| Maternal education (ref=none) |  |  |  |
| Primary graduate or less | 0.12 (0.03) | 0.15 (0.04) | 0.07 (0.05) |
| Secondary graduate or less | 0.17 (0.03) | 0.23 (0.04) | 0.06 (0.04) |
| College or above | 0.23 (0.03) | 0.38 (0.05) | 0.12 (0.05) |
| Type or residency (ref=rural) |  |  |  |
| Urban | 0.10 (0.02) | 0.22 (0.03) | -0.04 (0.03) |
| Caste (ref=scheduled tribe) |  |  |  |
| Scheduled caste | -0.20 (0.03) | -0.22 (0.05) | -0.10 (0.06) |
| Other backward class | -0.06 (0.02) | -0.03 (0.03) | -0.08 (0.04) |
| Others | 0.09 (0.03) | 0.14 (0.04) | 0.07 (0.04) |
| Wealth level (ref=1^st^ quintile (poorest)) |  |  |  |
| 2^nd^ quintile | 0.14 (0.03) | 0.14 (0.04) | -0.08 (0.06) |
| 3^rd^ quintile | 0.4 (0.03) | 0.40 (0.04) | 0.06 (0.06) |
| 4^th^ quintile | 0.52 (0.04) | 0.56 (0.05) | 0.12 (0.06) |
| 5^th^ quintile (richest) | 0.54 (0.04) | 0.67 (0.06) | 0.18 (0.06) |
| Paternal education (ref=none) |  |  |  |
| Primary graduate or less | 0.08 (0.09) | 0.14 (0.12) | 0.02 (0.15) |
| Secondary graduate or less | 0.12 (0.07) | 0.28 (0.1) | -0.05 (0.12) |
| College or above | 0.03 (0.08) | 0.18 (0.12) | -0.11 (0.12) |
| No paternal survey | 0.07 (0.06) | 0.19 (0.09) | -0.07 (0.11) |
| ***Health and medical factors*** |  |  |  |
| Low Birth Weight |  |  |  |
| Yes (<2,500 grams) | 0.12 (0.02) | 0.12 (0.03) | 0.13 (0.04) |
| Missing (Not recorded/Unknown) | -0.37 (0.04) | -0.15 (0.06) | -0.71 (0.06) |
| Baby size (ref=very small) |  |  |  |
| Smaller than average | -0.10 (0.05) | -0.04 (0.08) | -0.07 (0.08) |
| Average | -0.11 (0.05) | -0.06 (0.08) | -0.05 (0.07) |
| Larger than average | 0.05 (0.05) | 0.09 (0.08) | 0.08 (0.08) |
| Very large | 0.15 (0.06) | 0.17 (0.09) | 0.20 (0.09) |
| Mother’s height (ref=<145cm) |  |  |  |
| 145 -149 | -0.32 (0.03) | -0.31 (0.04) | -0.35 (0.05) |
| 150 -154 | -0.52 (0.03) | -0.52 (0.04) | -0.54 (0.04) |
| 155 -159 | -0.60 (0.03) | -0.58 (0.04) | -0.63 (0.05) |
| >=160 | -0.62 (0.04) | -0.55 (0.05) | -0.70 (0.05) |
| BMI (ref=<16 .0kg/m^2^) |  |  |  |
| 16.0 -18.4 | -0.01 (0.05) | 0.04 (0.08) | 0.01 (0.08) |
| 18.5 -24.9 | 0.23 (0.05) | 0.30 (0.07) | 0.21 (0.08) |
| 25.0 -29.9 | 0.73 (0.05) | 0.84 (0.08) | 0.67 (0.08) |
| >=30.0 | 1.11 (0.06) | 1.26 (0.09) | 1.03 (0.09) |
| Smoking (ref=no) |  |  |  |
| Yes | 0.28 (0.09) | 0.26 (0.12) | 0.56 (0.18) |
| Chewing Tobacco (ref=no) |  |  |  |
| Yes | -0.01 (0.04) | 0.01 (0.05) | -0.04 (0.07) |
| Alcohol (ref=no) |  |  |  |
| Yes | -0.16 (0.07) | -0.16 (0.09) | -0.14 (0.13) |
| Miscarriage, abortion or stillbirth (ref=no) |  |  |  |
| Yes | 0.14 (0.02) | 0.16 (0.03) | 0.12 (0.03) |
| ***Institutional factors*** |  | | |
| Insurance (ref=not covered) |  |  |  |
| Covered | 0.06 (0.02) | 0.05 (0.03) | 0.11 (0.04) |
| Place of delivery (ref=public) |  |  |  |
| Private | 1.46 (0.02) | - | - |
| ANC more than 4 times (ref=no) |  |  |  |
| Yes | 0.24 (0.02) | 0.22 (0.03) | 0.22 (0.03) |
| Notes: Fixed effects (logit) parameter estimates presented above derived from multilevel logistic regression models. M0 refers to intercept-only (null) model; M1 refers to models fully adjusted for sociodemographic + medical + institutional factors. | | | |

Table S4. Results from multiple imputation analysis: Population variance in cesarean delivery and variance explained among 146,713 women in India in 2016 comparing null (M0) to fully adjusted (M1) models, overall and by the type of delivery facility.

| **Group level** | **Overall** | | **Public** | | **Private** | |
| --- | --- | --- | --- | --- | --- | --- |
|  | **M0** | **M1** | **M0** | **M1** | **M0** | **M1** |
| ***State*** |  |  |  |  |  |  |
| Variance estimate (SE*) | 0.41 (0.11) | 0.24 (0.08) | 0.57 (0.08) | 0.32 (0.06) | 0.32 (0.07) | 0.26 (0.07) |
| Variance explained (%) | - | 41 | - | 44 | - | 19 |
| ***District*** |  |  |  |  |  |  |
| Variance estimate (SE*) | 0.23 (0.02) | 0.11 (0.01) | 0.26 (0.07) | 0.16 (0.05) | 0.16 (0.06) | 0.13 (0.05) |
| Variance explained (%) | - | 52 | - | 38 | - | 19 |
| ***Community*** |  |  |  |  |  |  |
| Variance estimate (SE*) | 0.31 (0.003) | 0.16 (0.002) | 0.28 (0.07) | 0.19 (0.05) | 0.16 (0.06) | 0.12 (0.05) |
| Variance explained (%) | - | 48 | - | 32 | - | 25 |
| Notes: M0: Null model / M1: Adjusted for all individual characteristics (20 for overall, 19 for public and private)  *SE: Standard error | | | | | | |

Figure S1. Results from multiple imputation analysis: Partitioning geographic variation in cesarean delivery among 146,713 women in India in 2016, overall and by the type of delivery facility.
